# Availability, timeliness, documentation and quality of consultations among hospital departments: a prospective, comparative study

**DOI:** 10.1186/s13584-021-00446-0

**Published:** 2021-04-19

**Authors:** Amir Jarjou’i, Joseph Mendlovic, Ziv Dadon, Marwan Abu Sneineh, Meir Tabi, George Kalak, Yousef R. Jarallah, Amos M. Yinnon, Gabriel Munter

**Affiliations:** 1 Internal Medicine, Shaare Zedek Medical Center, Affiliated with the Hadassah-Hebrew University School of Medicine, Jerusalem, Israel; 2 Division of Internal Medicine, Shaare Zedek Medical Center, Affiliated with the Hadassah-Hebrew University School of Medicine, P.O. Box 3235, 91031 Jerusalem, Israel; 3 Deputy CEO, Shaare Zedek Medical Center, Affiliated with the Hadassah-Hebrew University School of Medicine, Jerusalem, Israel; 4 Department of Cardiology, Shaare Zedek Medical Center, Affiliated with the Hadassah-Hebrew University School of Medicine, Jerusalem, Israel; 5 Department of Obstetrics and Gynecology, Shaare Zedek Medical Center, Affiliated with the Hadassah-Hebrew University School of Medicine, Jerusalem, Israel

**Keywords:** Consultations, Internal medicine, General surgery, Intensive care unit

## Abstract

**Background:**

Many in-patients require care from practitioners in various disciplines. Consultations most probably have significant implications for hospitalization outcomes.

**Purpose:**

To determine key aspects of consultations provided by various departments to formulate an optimal policy.

**Methods:**

This study comprised two methods: first, a questionnaire was completed in 2019 by 127 physicians interns, residents and senior doctors) from the medical and surgical departments (64 from the surgical wards, 43 from the medical wards and 22 from the emergency room and General ICU) regarding the availability, timeliness and documentation rate of the consultations they received from different disciplines. The investigators rounded through the various departments that were included in the study and they accosted a sample of interns, residents and attending physicians, who were then asked to fill the questionnaire. Overall compliance of filling the questionnaire was 95%. Residents accounted for 72% of the filled questionnaires, seniors and interns accounted for 15 and 13% respectively. Second, a convenience sample of 300 electronic records of hospitalized patients (135 from the surgical wards, 129 from the Medical wards and 36 from the emergency room and General ICU) of actually carried out consultations was reviewed for validated indicators of quality for both the consultation request and response. We used a 5-point Likert scale, ranging from poor (1) to superb (5), to grade the measured parameters.

**Results:**

The availability, timeliness and documentation rate for medical consultations were 4 ± 0.9, 4.1 ± 0.9 and 4.3 ± 0.9 respectively, as compared with surgical consultations 3.2 ± 1.1, 3.4 ± 1.2 and 3.6 ± 1.2 respectively (*P* < 0.001). The mean time (in hours) from the consultation request till documentation (of the requested consultation) by consultants in the medical and surgical departments was 3.9 ± 5.9 and 10.0 ± 15.6, respectively (*P* < 0.001). The quality of requests of consultations from the medical and surgical departments was 3.4 ± 1.1 and 2.8 ± 1.2, respectively (P < 0.001). Two different models of consultations are employed: while each medical department adopts several departments for medical consultations, each day’s on-call surgeon provides all the hospital’s surgical consultations.

**Conclusion:**

We detected significant differences in key aspects of consultations provided by the departments. The medical model of consultations, in which each medical department adopts several other wards to which it provides consulting services upon request, should probably be adopted as a major policy decision by hospitals directors to enhance inter-departmental consultations.

**Supplementary Information:**

The online version contains supplementary material available at 10.1186/s13584-021-00446-0.

## Introduction

The practice of medicine increasingly involves consultations with specialists in various subspecialties, accentuating the need for an in-charge coordinator [1–6]. The latter role is usually filled by the family practitioner in outpatient care and by the primary attending physician or surgeon in hospital-based medicine. For hospital-based physicians in the USA, the fee-for-service system has led to timely and quality consultations, albeit the associated spiraling costs have led to administrative curbs [7–9]. Various alternative systems have been experimented with to ensure available and good quality consultations, although at lower cost, without diminishing the motivation of consultants [10–13]. An ideal system, balancing between the various forces is evidently still out.

Each consultation starts with a request which ideally should be brief, precise, include relevant medical data, include caregiver contact information, state the urgency of the consultation and most importantly should include a well-defined question [14]. The consultant should answer the posed question in a clear and specific manner providing a brief list of recommendations and contingency plans if possible. The consultation should be performed within an acceptable time limit depending on the urgency and should be recorded in the patients’ electronic records [15, 16].

In the Israeli hospital system, consultations are provided by the various services without compensation, neither for the consultant nor for their department. Accordingly, up to a certain number of consultations the service can be expected to timely deliver quality consultations. However, as demand for consultations increases, beyond a certain number of consultations the providing services have been observed to become less available and willing to provide the necessary consultations. Both the requesting and providing physicians become frustrated, the former because they need to repeatedly submit reminders and receive evasive responses, and the latter because they feel cheap and abused. There is evidently need for reform. In this study we collected information on the timeliness and quality of consultations as delivered between the various hospital units. The accumulated data should serve as basis for an adaptation of the current system. We envision an improved system in which departments will receive positions to employ physicians, which also takes into account the number and frequency of provided consults per unit of time.

## Methods

The study was conducted in a 1000-bed general teaching hospital in Jerusalem. There are medical departments, surgical departments and Intensive Care Units for adults and children. The study did not include pediatric departments.

### Aim

The purpose of the current study was the assessment of the availability, timeliness and quality of consultations among departments. This study did not assess the availability, timeliness and quality of tests and imaging studies, such as done in the radiology department, echocardiograms in the cardiology department, gastroscopy/colonoscopy in the gastroenterology institute, lung function tests in the pulmonology institute, electro-encephalogram in the neurology institute, etc.

### Participating departments and physicians

Medical wards (162 physicians), Surgical wards (173 physicians) and General Intensive Care Unit (7 physicians). Medical Wards included: Internal Medicine (A, B, C, D), Geriatrics (A + B), Cardiology, Gastroenterology, Infectious Diseases, Pulmonology, Rheumatology, Endocrinology, Hematology, Nephrology, Stroke Unit, Psychiatry and Neurology. Surgical Wards included: General Surgery, General Orthopedics, Foot Orthopedics, Plastic Surgery, Otorhinolaryngology (ENT), Obstetrics and Gynecology, Urology, Cardiothoracic surgery, Neurosurgery and Ophthalmology.

### Endpoints

The major measurable endpoints were the availability, timeliness and documentation rates of consultations and the time from consultation request till documentation. Minor measurable endpoints were the quality of consultation requests and the quality of the made consultations. We used the 5 point Likert scale to quantitate the results where 1 indicated poor result and 5 an excellent result.

### Design of the study

#### Prospective and comparative

The study consisted of two components, a questionnaire and an evaluation of consults as documented in the electronic health record (EHR). The time span of the study was 8 months, ensuring inclusion of a large range of junior and senior physicians and surgeons.

#### First, the questionnaire component

The questionnaires were filled by a sample of interns, residents and senior doctors regarding the availability, timeliness and documentation rate of the different consult-providing disciplines. This was carried out in the following way:

1. The investigators rounded through the various departments that were included in the study, 2–3 times each week. In addition, they joined the relevant departments’ staff meetings, or early morning rounds.
2. They accosted a sample of interns, residents and attending physicians, who were the most likely staff to request consultations.
3. The latter were requested to participate in the study in order to evaluate the management of consultations in the hospital. Their cooperation was anonymous and required less than 5 min. Participants were obliged to fill the questionnaire on the spot and return it to the investigator in a blank envelop or box (For the questionnaires see Additional file 1).

#### Second, the record component

The patient electronic health records (EHR) were reviewed and a sample of provided consultations was examined for validated indicators of quality. The investigators reviewed the files of admitted patients in each of the departments which requested consultations in order to include a sample of the consultations. This was a convenience sample from consultations made during the morning shift on working days and did not include curbside, undocumented, telephone consultations and consultations provided on weekends/holidays. More than one request per patient was allowed. The data were entered in a specific questionnaire (Additional file 2).

#### The questionnaires where anonymous

The directors of all included departments, at the start of the study, provided written consent to participate in this study. The conduct of the study was approved by the local Internal Review Board (Helsinki committee). The study received funding from the Mirsky Foundation.

#### Statistics

Data analysis was performed for each consulting department, but also for three blocks, Medical wards, Surgery wards and General Intensive care unit.

Data were entered into an Excel spreadsheet. Statistical analysis was done with SPSS 20th version. For categorical variables we employed Chi square analysis or Fisher exact test, as required. For continuous variables we used the t-test or non-parametric tests, as needed. When necessary, the Mann-Whitney test was used. A *P* value of < 0.01 was considered statistically significant.

## Results

**Fig. 1:**
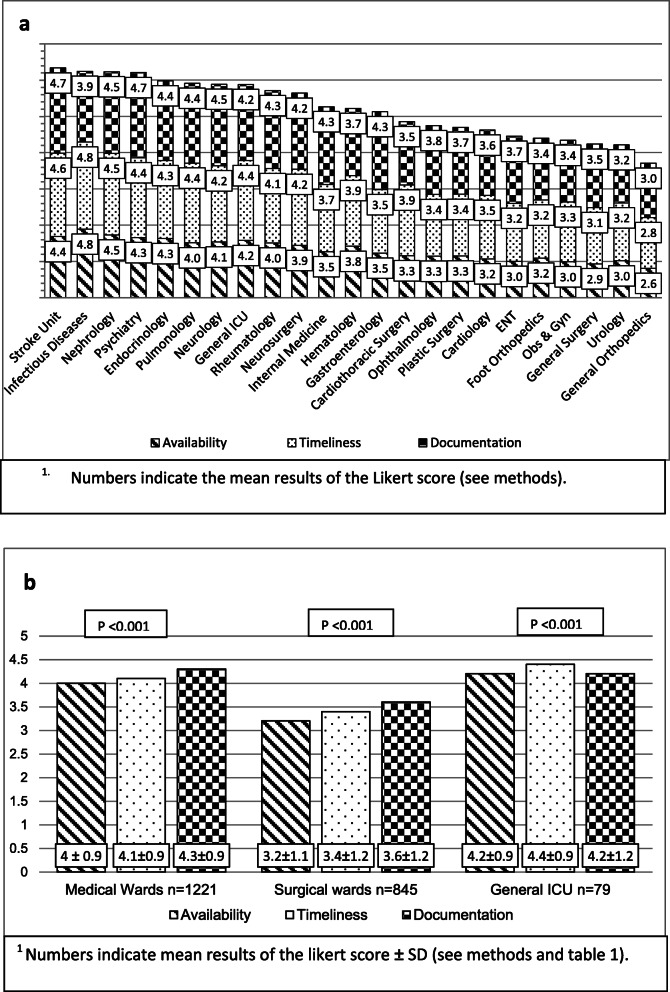
**a**: Availability, timeliness and documentation of consult-providing diciplines^1^. **b**: Availability, timeless and documentation of wards providing consultations^1^

As described previously the study included two components. The first component consisted of a questionnaire that was used to assess the availability, timeliness and documentation of consultations as perceived by interns, residents and senior doctors, and they scored each department from 1 to 5. For the medical wards, 1221 questionnaires were completed, for the surgical wards 845 questionnaires were completed and 79 questionnaires for the general Intensive Care Unit (Table 1). Overall compliance of filling the questionnaire was 95%. Residents accounted for 72% of the filled questionnaires, seniors and interns accounted for 15 and 13% respectively. There were significant differences for all three parameters between the 3 divisions as well as for individual departments (Fig. 1a+b).

**Table 1:** Availability, timeliness and documentation of departments providing consultations^a^

	Medical consultations^b^ *N* = 1221	Surgical consultations *N* = 845	General ICU consultations *N* = 79	*P* value
Availability	4.0 ± 0.9	3.2 ± 1.1	4.2 ± 0.9	<0.001
Timeliness	4.1 ± 0.9	3.4 ± 1.2	4.4 ± 0.9	<0.001
Documentation	4.3 ± 0.9	3.6 ± 1.2	4.2 ± 1.2	<0.001

^a^Results shown as Mean score ± SD (range 1–5)

^b^For definition refer to methods

**Fig. 2:**
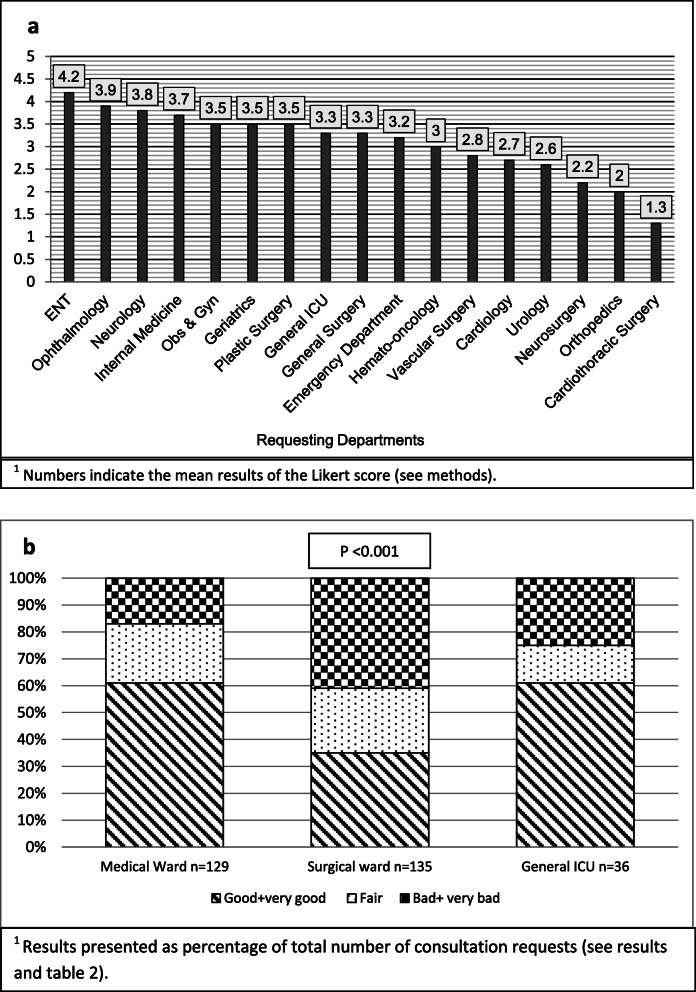
**a**: Quality of requests of consultations (mean score out of 5)^1^. **b**: Quality of requests of consultations (mean score out of 5)^1^

**Fig. 3:**
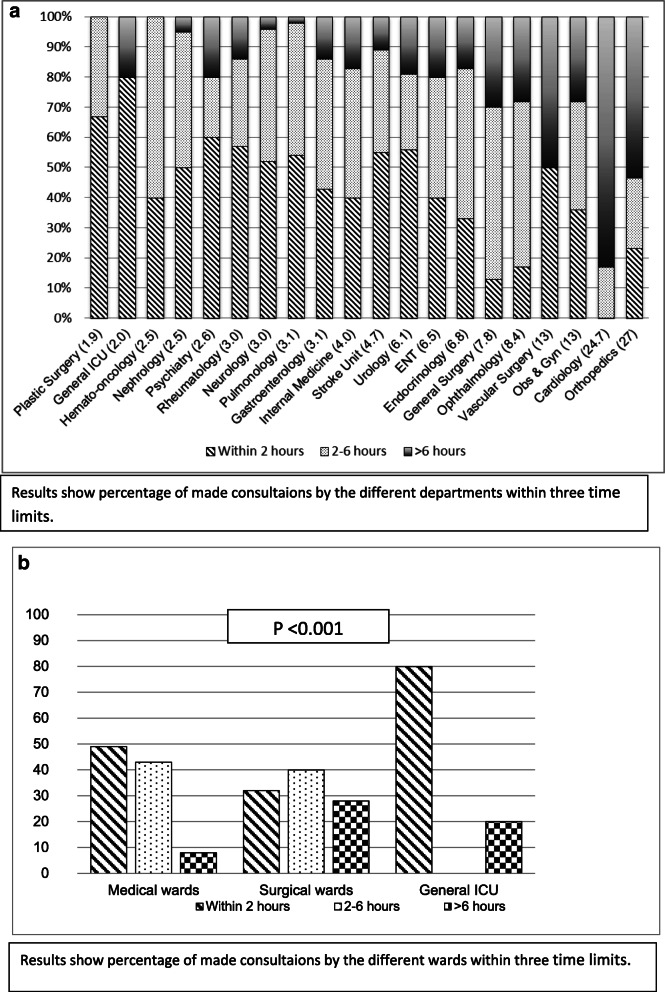
**a**: Timelines of made consultations (%). **b**: Timeless of made consultations (%)

In the second part, EHRs of made consultations were reviewed for validated indicators of quality for both the consultation requests and responses. For the medical wards 129 records of consultation requests were assessed with a mean score of 3.4 ± 1.1. For the surgical wards 135 consultation requests were assessed and the mean score was 2.8 ± 1.2. For the general ICU 36 requests were assessed with a mean score of 3.3 ± 1.1 (*P* < 0.001) (Table 2). The percentage of good-very good requests was 61% for the medical wards and for ICU requests, while in comparison only 35% for surgical wards (*P* < 0.001) (Table 2). The differences between the specific departments were also obvious (Fig. 2a+b). The made consultations were also assessed for timeliness and quality (Table 3). The time from the consultation request till documentation of made consultations was highly variable between the consulting departments (Fig. 3a+b). There were no significant differences in the quality of consultations made between different wards; the follow up rate was low across the board (Table 3).

**Table 2:** Quality of requests for consultations

	Medical wards^b^ *N* = 129	Surgical wards *N* = 135	General ICU *N* = 36	*P* value
1. Mean score ± SD	3.4 ± 1.1	2.8 ± 1.2	3.3 ± 1.1	<0.001
2. Quality assessment^a^	< 0.001			
Good+ very good (4/5 + 5/5)	79 (61%)	47 (35% (	22 (61%)	
Fair (3/5)	28 (22%)	32 (24%)	5 (14%)	
Bad+Very bad (2/5–1/5)	22 (17%)	56 (41%)	9 (25%)	

^a^Results shown as n(%)

^b^For definition refer to methods

**Table 3:** Timeliness and quality of made consultations

	Medical wards^b^ *N* = 186	Surgical wards *N* = 109	General ICU *N* = 5	*P* value
A. Time till consult in hours^a^	3.9 ± 5.9	10.0 ± 15.6	2.0 ± 2.5	<0.001
B. Did the consultation include: (% of relevant)				
1. Clear answer to the request	100%	97%	100%	NS
2. Relevant history	94%	83%	80%	
3. Physical examination	91%	88%	80%	
4. Review of relevant tests	97%	84%	80%	
5. Discussion	95%	84%	80%	
6. Recommendations	100%	99%	89%	
7. Follow up	37%	22%	0%	

^a^Results shown as (mean ± SD)

^b^For definition refer to methods

On average 56 consultations are provided by the Medical wards each day, 28 are provided by the Surgical wards and 1 by General ICU. There are a total of 162, 173, and seven senior physicians and residents in the Medical wards, Surgical wards and General ICU respectively.

## Discussion

Consultations are an integral part of the daily routine in hospitals. In a non-fee-for-service system, the efficiency of consultations can be affected by several factors, especially the readiness and availability of the consultants [17].

This study documented the differences in consultation services provided mainly by the medical and surgical disciplines.

The following were the major findings: Significant differences in the availability, timeliness and documentation rate between the two disciplines are apparent. Twice as many consultations are provided by the medical wards in comparison with the surgical wards despite having a similar total number of physicians. Significant differences were also seen between specific departments. The time interval between the consultation request until documentation of the provided consult was also significantly different between the various wards and between specific departments. The quality of consultation requests was highly variable between the different departments; this has major impact on the consultants’ understanding of the request and its urgency.

We detected a major difference in the approach and organization of the consultation service between the medical and surgical departments. In the surgical wards, mainly general surgery and orthopedics, the on-call surgeon for a 24 h period is requested to provide all consultations upon request to various consult-requesting departments. This person, however, has additional responsibilities such as operating, out-patient clinics, etc. In comparison, the medical consulting service is organized differently; each medical department adopts several other wards to which it provides consulting services upon request. This arrangement automatically assumes responsibility for timeliness and availability: at least one of several attending physicians will be able to provide urgent consultations. In the surgical disciplines, the consult providing service is deemed the responsibility of one person (who has additional functions) without departmental back-up. Moreover, whereas the surgical approach does not provide for continuity (as each 24 h another surgeon is on-call), the medical department inherently allows for continuity by the same attending, who, if unable to return to the patient, has a higher sense of obligation to transfer the relevant clinical information to another attending in his/her department.

Ultimately, in the medical approach to consultations, once the medical department receives a request for a consultation it accepts responsibility to dispatch (as early as possible and needed) an attending to perform the consultation. Whereas in the surgical approach, the responsibility for locating, contacting and obtaining a surgical consultant (or a replacement if he/she is busy) is thrown back to the physician whose patient is in need of a surgical consultation. This obviously needs repair.

It remains to be determined what the best consultation service looks like. The timeliness of consultations is highly dependent on the situation and urgency. Although a 2 h delay on a regular consult may appear adequate, this might be too long for urgent cases. On one hand, it is obvious that the consult-requesting physicians would always appreciate a quicker consultation. On the other hand, a reasonable time limit should be considered. We believe that urgent consultations should be provided within minutes up to 1-h, most consultations should be provided within a 2-h limit, up to 6 h can be acceptable. Consultations provided after 6 h may delay considerably patient management plans. The results of the first component of the study, the questionnaire, clearly show that the physicians ordering the consultations also believe that there is much room for improvement in most departments.

The consultation service should be improved in all the departments included in our study, for which we have several suggestions [18]. First, the medical department approach should be adopted rather than the individual surgical one [19]. Second, hospitals should implement a reward system for the departments after a certain number of consultations made per month. As a result, understaffed units will, obviously, benefit from an increase in staff members. Third, if possible, one of the staff should be responsible for the consultation service as his main task instead of it being an add-on or nominating a permanent consultant for specific periods of time could improve availability and timeliness. Fourth, using a computerized notification regarding consultation requests could also improve availability and timeliness [20, 21]. Fifth, the quality of requests of consultations should also be increased, which can improve both the communication with the consultant and his understanding of the nature of the request [22].

It is obvious that the changes need to be implemented by hospital directors, and possibly the Ministry of Health. Consultations are an integral part of the patients’ management and care during hospitalization and should be considered as crucial as any other daily duties. The Ministry of Health should issue an official change of policy emphasizing changes needed to improve the consultation service.

### Limitations

This study has several limitations. First, this was a single center study which did not include pediatric departments. Second, consultations not documented in the medical records could not be assessed for quality, timeliness or percentage of all the consultations. Third, the study did not include overall number of consultations made per department, and included only consultations done during the weekdays and during the morning shifts to minimize the impact of lack of personnel on the study results. However, weekends and night shifts are an important part of the services provided and another study should be considered to assess these personnel-scarce times. With that in mind, it is reasonable to conclude that during night and weekend shifts, the consultation services are not superior or faster than during morning and weekday shifts, when the presence of staff members is the highest. Fourth, and possibly the major limitation of this study is a lack of a post intervention phase. Although, initially planned, it required administration-led interventions as outlined previously. This study’s data were presented to the hospital’s administration who expressed appreciation for the study and its findings. Plans for presenting the data to the departments’ directors have so far not been implemented due to various reasons, including the COVID-19 epidemic.

## Conclusion

The consultation service can be improved by using several policies; it should be regarded as an integral part of the daily tasks and not as an add-on service. First, we recommend organization of consultations along the medical departments’ model, rather than the surgical “one person responsibility” approach. Second, we suggest including number of consultations made when calculating staff members needed. Third, we suggest using a reward system of the departments depending on the amount of the consultations made. Fourth, we suggest tasking members with the specific responsibility of providing consultations which should improve efficiency. Fifth, we suggest improving the quality of consultation requests by the use of a structured request, which should improve the consultants’ understanding of the requested consultation and its urgency.

## Supplementary Information

**Additional file 1.**

**Additional file 2.**

## Data Availability

All used questionnaires and all accumulated data are included in the manuscript.
