# Supplementary material for: Availability, timeliness, documentation and quality of consultations among hospital departments: a prospective, comparative study
Source: Isr J Health Policy Res. 2021 Apr 19;10:19. doi: 10.1186/s13584-021-00446-0 (PMC8053423; doi:10.1186/s13584-021-00446-0)
Supplement: Supplementary file 1 — Additional file 1. [file 13584_2021_446_MOESM1_ESM.docx]

**Appendix A: Consultant's availability, timeliness and documentation score**

1. ***Availability***

| **Unknown** | **Very High**  **- 5 -** | **High**  **- 4 -** | **Moderate**  **- 3 -** | **Low**  **- 2 -** | **Very low**  **- 1 -** | **Department \| Score** |
| --- | --- | --- | --- | --- | --- | --- |
| 🞎 | ➄ | ➃ | ➂ | ➁ | ➀ | 1. Infectious diseases |
| 🞎 | ➄ | ➃ | ➂ | ➁ | ➀ | 1. Gastroenterology |
| 🞎 | ➄ | ➃ | ➂ | ➁ | ➀ | 1. Cardiology |
| 🞎 | ➄ | ➃ | ➂ | ➁ | ➀ | 1. Nephrology |
| 🞎 | ➄ | ➃ | ➂ | ➁ | ➀ | 1. Pulmonology |
| 🞎 | ➄ | ➃ | ➂ | ➁ | ➀ | 1. Rheumatology |
| 🞎 | ➄ | ➃ | ➂ | ➁ | ➀ | 1. Hematology |
| 🞎 | ➄ | ➃ | ➂ | ➁ | ➀ | 1. Endocrinology |
| 🞎 | ➄ | ➃ | ➂ | ➁ | ➀ | 1. Internal Medicine and Geriatrics |
| 🞎 | ➄ | ➃ | ➂ | ➁ | ➀ | 1. Psychiatry |
| 🞎 | ➄ | ➃ | ➂ | ➁ | ➀ | 1. Neurology |
| 🞎 | ➄ | ➃ | ➂ | ➁ | ➀ | 1. Stroke Unit |
| 🞎 | ➄ | ➃ | ➂ | ➁ | ➀ | 1. General Surgery |
| 🞎 | ➄ | ➃ | ➂ | ➁ | ➀ | 1. General Orthopedics |
| 🞎 | ➄ | ➃ | ➂ | ➁ | ➀ | 1. Foot Orthopedics |
| 🞎 | ➄ | ➃ | ➂ | ➁ | ➀ | 1. Plastic Surgery |
| 🞎 | ➄ | ➃ | ➂ | ➁ | ➀ | 1. Urology |
| 🞎 | ➄ | ➃ | ➂ | ➁ | ➀ | 1. ENT |
| 🞎 | ➄ | ➃ | ➂ | ➁ | ➀ | 1. Ophthalmology |
| 🞎 | ➄ | ➃ | ➂ | ➁ | ➀ | 1. Obstetrics and Gynecology |
| 🞎 | ➄ | ➃ | ➂ | ➁ | ➀ | 1. Neurosurgery |
| 🞎 | ➄ | ➃ | ➂ | ➁ | ➀ | 1. Cardio-Thoracic surgery |
| 🞎 | ➄ | ➃ | ➂ | ➁ | ➀ | 1. General Intensive Care Unit |

Please go through the following list of consultants and score the ***Availability*** (reachability). In case you don’t have enough experience with any of them please mark as unknown.

1. ***Timeliness***

Please go through the following list of consultants and score the ***Timeliness*** (time till consultation). In case you don’t have enough experience with any of them please mark as unknown.

| **Unknown** | **Very High**  **- 5 -** | **High**  **- 4 -** | **Moderate**  **- 3 -** | **Low**  **- 2 -** | **Very low**  **- 1 -** | **Department \| Score** |
| --- | --- | --- | --- | --- | --- | --- |
| 🞎 | ➄ | ➃ | ➂ | ➁ | ➀ | 1. Infectious diseases |
| 🞎 | ➄ | ➃ | ➂ | ➁ | ➀ | 1. Gastroenterology |
| 🞎 | ➄ | ➃ | ➂ | ➁ | ➀ | 1. Cardiology |
| 🞎 | ➄ | ➃ | ➂ | ➁ | ➀ | 1. Nephrology |
| 🞎 | ➄ | ➃ | ➂ | ➁ | ➀ | 1. Pulmonology |
| 🞎 | ➄ | ➃ | ➂ | ➁ | ➀ | 1. Rheumatology |
| 🞎 | ➄ | ➃ | ➂ | ➁ | ➀ | 1. Hematology |
| 🞎 | ➄ | ➃ | ➂ | ➁ | ➀ | 1. Endocrinology |
| 🞎 | ➄ | ➃ | ➂ | ➁ | ➀ | 1. Internal Medicine and Geriatrics |
| 🞎 | ➄ | ➃ | ➂ | ➁ | ➀ | 1. Psychiatry |
| 🞎 | ➄ | ➃ | ➂ | ➁ | ➀ | 1. Neurology |
| 🞎 | ➄ | ➃ | ➂ | ➁ | ➀ | 1. Stroke Unit |
| 🞎 | ➄ | ➃ | ➂ | ➁ | ➀ | 1. General Surgery |
| 🞎 | ➄ | ➃ | ➂ | ➁ | ➀ | 1. General Orthopedics |
| 🞎 | ➄ | ➃ | ➂ | ➁ | ➀ | 1. Foot Orthopedics |
| 🞎 | ➄ | ➃ | ➂ | ➁ | ➀ | 1. Plastic Surgery |
| 🞎 | ➄ | ➃ | ➂ | ➁ | ➀ | 1. Urology |
| 🞎 | ➄ | ➃ | ➂ | ➁ | ➀ | 1. ENT |
| 🞎 | ➄ | ➃ | ➂ | ➁ | ➀ | 1. Ophthalmology |
| 🞎 | ➄ | ➃ | ➂ | ➁ | ➀ | 1. Obstetrics and Gynecology |
| 🞎 | ➄ | ➃ | ➂ | ➁ | ➀ | 1. Neurosurgery |
| 🞎 | ➄ | ➃ | ➂ | ➁ | ➀ | 1. Cardio-Thoracic surgery |
| 🞎 | ➄ | ➃ | ➂ | ➁ | ➀ | 1. General Intensive Care Unit |

1. ***Documentation rate***

Please go through the following list of consultants and score the ***Documentation rate***. In case you don’t have enough experience with any of them please mark as unknown.

| **Unknown** | **Very High**  **- 5 -** | **High**  **- 4 -** | **Moderate**  **- 3 -** | **Low**  **- 2 -** | **Very low**  **- 1 -** | **Department \| Score** |
| --- | --- | --- | --- | --- | --- | --- |
| 🞎 | ➄ | ➃ | ➂ | ➁ | ➀ | 1. Infectious diseases |
| 🞎 | ➄ | ➃ | ➂ | ➁ | ➀ | 1. Gastroenterology |
| 🞎 | ➄ | ➃ | ➂ | ➁ | ➀ | 1. Cardiology |
| 🞎 | ➄ | ➃ | ➂ | ➁ | ➀ | 1. Nephrology |
| 🞎 | ➄ | ➃ | ➂ | ➁ | ➀ | 1. Pulmonology |
| 🞎 | ➄ | ➃ | ➂ | ➁ | ➀ | 1. Rheumatology |
| 🞎 | ➄ | ➃ | ➂ | ➁ | ➀ | 1. Hematology |
| 🞎 | ➄ | ➃ | ➂ | ➁ | ➀ | 1. Endocrinology |
| 🞎 | ➄ | ➃ | ➂ | ➁ | ➀ | 1. Internal Medicine and Geriatrics |
| 🞎 | ➄ | ➃ | ➂ | ➁ | ➀ | 1. Psychiatry |
| 🞎 | ➄ | ➃ | ➂ | ➁ | ➀ | 1. Neurology |
| 🞎 | ➄ | ➃ | ➂ | ➁ | ➀ | 1. Stroke Unit |
| 🞎 | ➄ | ➃ | ➂ | ➁ | ➀ | 1. General Surgery |
| 🞎 | ➄ | ➃ | ➂ | ➁ | ➀ | 1. General Orthopedics |
| 🞎 | ➄ | ➃ | ➂ | ➁ | ➀ | 1. Foot Orthopedics |
| 🞎 | ➄ | ➃ | ➂ | ➁ | ➀ | 1. Plastic Surgery |
| 🞎 | ➄ | ➃ | ➂ | ➁ | ➀ | 1. Urology |
| 🞎 | ➄ | ➃ | ➂ | ➁ | ➀ | 1. ENT |
| 🞎 | ➄ | ➃ | ➂ | ➁ | ➀ | 1. Ophthalmology |
| 🞎 | ➄ | ➃ | ➂ | ➁ | ➀ | 1. Obstetrics and Gynecology |
| 🞎 | ➄ | ➃ | ➂ | ➁ | ➀ | 1. Neurosurgery |
| 🞎 | ➄ | ➃ | ➂ | ➁ | ➀ | 1. Cardio-Thoracic surgery |
| 🞎 | ➄ | ➃ | ➂ | ➁ | ➀ | 1. General Intensive Care Unit |

- Professional status : ⭘Intern ⭘ Resident ⭘ Senior Physician
- Department :_____________
