# Supplementary material for: Availability, timeliness, documentation and quality of consultations among hospital departments: a prospective, comparative study
Source: Isr J Health Policy Res. 2021 Apr 19;10:19. doi: 10.1186/s13584-021-00446-0 (PMC8053423; doi:10.1186/s13584-021-00446-0)
Supplement: Supplementary file 2 — Additional file 2. [file 13584_2021_446_MOESM2_ESM.docx]

Appendix B: Objective assessment of consultations request and response for quality

File #:………….

Demographic information:

1. Department that requested the consultation:…………………
2. Identity of department/service that provided the consultation…………….
3. Consultation request quality : very good / good / fair / poor / very poor
4. Did the request include a clear question? Yes/no
5. Did the request include urgency? Yes/no
6. What was the urgency as specified in the request? Urgent / for today / regular
7. Did the request include relevant medical data? Yes/no

Consultation assessment:

1. Time interval between the requested and actually made consult:……hours
2. Short history. Yes/ no/
3. Short physical examination, yes /no /
4. Short review of relevant tests, yes/ no / not relevant
5. Discussion, yes/no/NA
6. Was the requesting physician's question answered? Yes / No / not relevant
7. Were recommendations made? Yes/no. If yes, were recommendations structured? Yes/ no
8. Was there a documented follow-up visit? Yes/ no/not relevant
